# Diet quality as assessed by Healthy Eating Index-2015 among Hungarian Roma living in settlements of Northeast Hungary

**DOI:** 10.1038/s41598-022-23670-3

**Published:** 2022-11-10

**Authors:** Helga Bárdos, Erand Llanaj, Ferenc Vincze, Judit Diószegi, Péter Pikó, Zsigmond Kósa, János Sándor, Róza Ádány

**Affiliations:** 1 Department of Public Health and Epidemiology, Faculty of Medicine, University of Debrecen, Kassai Street 26/B, Debrecen, 4028 Hungary; 2 MTA-DE Public Health Research Group, University of Debrecen, Kassai Street 26/B, Debrecen, 4028 Hungary; 3 Department of Methodology for Health Visitors and Public Health, Faculty of Health, University of Debrecen, Sóstói Street 2-4, Nyíregyháza, 4400 Hungary; 4 Department of Public Health, Semmelweis University, Budapest, Hungary

**Keywords:** Medical research, Risk factors

## Abstract

Inequalities in diet quality are increasingly reported, but such studies among Roma are scarce and challenging. Here we attempt to examine diet quality and adherence to food based dietary guidelines among Hungarian Roma (HR) ethnic minority living in segregated settlements while comparing a sample of Hungarian adults from the general population (HG). Data were obtained from a complex comparative health survey conducted in Northeast Hungary in 2018, including sociodemographic and physical examination data. Dietary data were collected using two non-consecutive 24-h dietary recalls. We assessed diet quality based on using a 13-component Healthy Eating Index-2015 (HEI-2015, range 0–100). Differences in median intakes of food and nutrients and HEI-2015 scores were evaluated by Mann–Whitney test or Kruskal–Wallis test. Quantile regression was used to adjust HEI-2015 scores for socioeconomic factors including age, sex, educational status, and perceived financial status. This analysis included 393 and 415 subjects, aged between 18 to 70 years, of HR and HG populations, respectively. Results showed overall low median HEI-2015 scores for both HR and HG, with significantly lower total score among HR participants (41.6, interquartile range (IQR): 39.5–42.8) compared to HG (47.2, IQR: 45.7–51.1). Scores for individual components, such as intake of fruits, greens and beans, whole grains, seafood, and plant proteins were particularly suboptimal among both groups, but significantly lower among the HR population. Scores for refined grains, sodium, saturated fats and added sugar reflected high intakes of these components but did not differ between study groups. Our findings revealed an unfavorable diet quality among the HR compared to HG and a potentially increased risk for diet-related NCDs. Future health intervention programs are warranted to address dietary disparities of segregated minorities in Hungary while considering ethnic and cultural differences.

## Introduction

Roma constitutes the largest ethnic minority in Europe with an estimated population of 11.3 million^1^ and Hungary has one of the largest share of Roma, i.e. more than 8% of the total population^2^. A considerable proportion of the Hungarian Roma (HR) population lives in segregated settlements, located mainly in rural areas or outskirts of cities in the Northeast part of the country. Living conditions in these settlements indicate an accumulation of socioeconomic disadvantages such as segregation, poor housing condition, overcrowding, waste deposits, pollution, and often an absence of basic services such as drinking water, sanitation, gas, and electricity^3^. These conditions pose a great risk for the health and wellbeing of these communities.

Therefore, health research involving Roma participants is imperative to inform decisions that address their health challenges. However, participation of this minority in health research has been traditionally hindered by difficulties emerging from defining their ethnicity and field data collection challenges^4–6^. Nevertheless, the available data that exists, indicate worse health status for the majority of Roma population, compared to that of the non-Roma population in Europe, independently of the country of residence^4,7–11^. In Central Europe, the prevalence of diet-related noncommunicable diseases (NCDs), such as type 2 diabetes (T2DM), metabolic syndrome, and cardiovascular diseases (CVDs) has been shown to be especially higher among Roma, as compared to the general population^8,12–14^.

In Hungary, a recent comparative risk assessment of 10-year CVD risk revealed up to three times higher CVD risk among the HR compared to the general population^15^. Intriguingly, CVD risk factors, including serum cholesterol levels, high blood pressure, insulin resistance, and obesity have been shown to be strongly correlated with dietary and nutrient intake patterns^16^. Results of a large multi-ethnic cohort study suggest that adhering to a dietary pattern that achieves a high diet-quality index score is associated with lower risk of mortality from all causes, CVDs, and cancer among adult men and women^17^. Several scoring systems have been developed for evaluating the quality of the diet according to recommended nutrient intake and dietary patterns^18–21^.

The traditional Hungarian diet is characterized by the consumption of high amounts of white bread and refined grain products, preserved vegetables, processed meat, and lard which is typical in Eastern European countries^22^. The Hungarian Diet and Nutritional Status Survey in 2014 revealed unfavorable changes compared to the previous survey in 2009, with an increase in fat and saturated fatty acid intake and a decrease in fruit and vegetable consumption^23^. Dietary data for HR have only been published recently by our research group. Based on recommendations defined by the World Health Organization, in the Dietary Approaches to Stop Hypertension (DASH) study and the EAT-Lancet report, as well as dietary quality based on Dietary Inflammatory Index (DII), we found substandard adherence to established international health and environmental sustainability guidelines in participants from HR settlements^24,25^.

Although informative and useful, our previous analyses have been restricted to nutrient-level evaluations, but no food-level assessment has been conducted. The latter can serve as a basis for food-based dietary guidelines, which is one of the predominant strategies for providing public advice on foods, food groups and dietary patterns, to promote a healthy diet and prevent diet-related NCDs^26–28^.

In this analysis, to the best of our knowledge, we examine for the first time the quality of diet and adherence to food-based nutrition guidelines using HEI-2015 on a sample of HR ethnic minorities living in segregated settlements, while comparing the results with that of the general population.

## Materials and methods

### Study design and data

All data used in the current analysis were obtained in a cross-sectional survey conducted between May and August 2018 in Northeast Hungary. This complex health (behavior and examination) survey collected questionnaire-based, physical and laboratory examination data. Details of sampling and data collection and management were described previously^13^.

In brief, HR populations living in segregated colonies in Northeast Hungary (Hajdú-Bihar and Szabolcs-Szatmár-Bereg counties) and HG populations from the same geographical area were randomly selected. In the sampling process, 25 Roma colonies with more than 100 inhabitants were selected. For each colony, 20 households were selected, and for each household, one adult person aged more than 18 years was interviewed. These interviews were conducted in the respondent’s households by Roma university students under the supervision of public health coordinators. Participant ethnicity was assessed by self-report. The HG population was chosen from 20 randomly selected general practitioners (GP), for each GP practice 25 randomly selected individuals aged more than 18 years were selected and interviewed by GP nurses. The planned sample size for both study groups was 500 and 500 participants, 85 individuals refused to participate in the study. For this analysis, we used data from only for those participants who had complete records (393 individuals of Roma settlements and 415 participants of HG sample).

Socio-demographic data were derived from the questionnaire-based part of the complex health survey and included age, sex, educational level, self-reported financial status. Educational level was categorized as elementary (8 years of primary school or less), secondary (high school general graduation), vocational (vocational school with certifying vocational qualifications), and university degree (diploma obtained in higher education, in college or university (BA/BSc, MA/MSc, PhD, DLA). Data on body mass index (BMI) and nutritional status were based on measured weight and height values. All measurements were carried out by GP nurses using validated equipment according to a standardized protocol as a part of the health examination survey in GP offices. The nutritional status of participants was categorized according to the cut-off points proposed by WHO^29^. Nutritional status was classified as underweight if BMI < 18.5 kg/m^2^, normal if BMI 18.5–24.9 kg/m^2^; overweight 25.0–29.9 kg/m^2^; and obese > 30.0 kg/m^2^.

Dietary intake data were obtained by an interview-assisted two-day (non-consecutive weekday and a weekend day) 24-h dietary recall (24hDR) assessment, the protocol of which was validated and published previously^30^. The interval between the first and second 24hDR assessment was between 2 and 14 days. Only those participants data were analyzed who had complete two-day records. Dietary intake was quantified and processed with NutriComp DietCAD ver. 3.03 software^31^ previously used in Hungarian Dietary and Nutritional Status surveys^23,32^. The software contains detailed food composition information on 1328 food items and 1823 recipes. Macro- and micronutrient intakes from each individual input of dietary intake data (e.g., food, meals, and drinks) were computed as a mean of the two 24hDRs. The present analysis used nutrient data of total energy intake, intake of saturated, mono- and poly-unsaturated fatty acids, added sugar, and sodium. Individual food intake data were manually collected, grouped, and counted according to the definition of Healthy Eating Index-2015 (HEI-2015) components^33,34^ from individual ingredients intake data, which were generated from inputs of dietary intake by NutriComp DietCAD ver. 3.03 software. Five participants who reported implausibly low energy intakes (females less than daily 700 kcal, males less than 800 kcal) were excluded from the analyses.

### Healthy Eating Index-2015 score

Diet quality was assessed by using HEI-2015 scores. HEI-2015 is a measure of diet quality commonly used to evaluate how well a set of foods aligns with the 2015–2020 Dietary Guidelines for Americans which is grounded on the current state of scientific evidence on nutrition and health, which reflects international scientific recommendations to promote better diet and reduce the risk of non-communicable diseases^33–35^.

The HEI-2015 includes 13 components that capture the balance among food groups including those to encourage (adequacy components) and those for which there are limits (moderation components). Higher scores of adequacy components reflect higher intakes that meet the standards. Higher scores of moderation components indicate lower intakes that are more desirable. Intakes between the minimum and maximum standards are scored proportionately.

Adequacy components include total fruits, whole fruits, total vegetables, greens and beans, whole grains, dairy products, total protein foods, and seafood and plant proteins. Total fruits component includes 100% fruit juice, the standard for maximum score of five is 0.8 cup equivalent or more per 1000 kcal and for minimum score of zero is no fruit intake. Whole fruits component includes all forms except juice, the standard for maximum score of five is 0.4 cup equivalent or more per 1000 kcal and for minimum score of zero is no whole fruit. Total vegetables component includes all vegetables and legumes (beans and peas), the standard for maximum score of five is 1.1 cup equivalent or more per 1000 kcal and for minimum score of zero is no vegetables. Greens and beans component includes legumes and dark-green leafy vegetables (i.e., salad greens like romaine lettuce, cruciferous cooking greens like kale and broccoli). The standard for maximum score of five is 0.2 cup equivalent or more per 1000 kcal and for minimum score of zero is no dark-green vegetables, beans, or peas. Whole grains component includes food made from whole grains (like wheat, mature corn, rice, oats, barley, quinoa, sorghum, spelt, and rye). The standard for maximum score of ten is 1.5 oz equivalent or more per 1000 kcal and for minimum score of zero is no whole grains. Diary component includes all milk products such as fluid milk, yogurt, cheese, and fortified soy beverages. The standard for maximum score of ten is 1.3 cup equivalent or more per 1000 kcal and for minimum score of zero is no diary. Total protein foods component includes all foods made from meat, poultry, seafood, beans and peas, eggs, processed soy products, nuts, and seeds. The standard for maximum score of five is 2.5 oz equivalent or more per 1000 kcal and for minimum score of zero is no protein foods. Seafood and plant proteins component includes seafood, nuts, seeds, soy products (other than beverages), and legumes (beans and peas). The standard for maximum score of five is 0.8 oz equivalent or more per 1000 kcal and for minimum score of zero is no seafood or plant proteins. Fatty acids component is a ratio of poly- and mono-unsaturated fatty acids (PUFAs and MUFAs) to saturated fatty acids (SFAs). The standard for maximum score of ten is 2.5 or more and for minimum score of zero is 1.2 or less.

Moderation components include refined grains, sodium, added sugar and saturated fatty acids. Refined grains component includes food made from refined grain (like white bread, pasta, and rice). The standard for maximum score of ten is 1.8 oz equivalent or less per 1000 kcal and for minimum score of zero is 4.3 oz equivalent or more per 1000 kcal. Sodium component calculated from salt content of daily food intake. The standard for maximum score of ten is 1.1 g or less per 1000 kcal and for minimum score of zero is 2.0 g or more per 1000 kcal. Added sugars component includes caloric sweeteners and syrups used as sweeteners in other food products, as well as sugars added in food preparation, processing, and added at the table. The standard for maximum score of ten is 6.5% of energy or less and for minimum score of zero is 26% of energy or more. Saturated fats component calculated from saturated fatty acids content of daily food intake. The standard for maximum score of ten is 8% of energy or less and for minimum score of zero is 16% of energy or more.

The amount of food was calculated individually for each food item using the Food Patterns Equivalents Database 2015–2016^36^.

### Data analysis

Food and nutrient intake data were compiled in Microsoft Excel software and matched to sociodemographic data. Differences in means or percentage distributions of sociodemographic characteristics were tested by t-test or chi-square test. The average intake of food and nutrients and HEI-2015 scores were presented as medians and accompanied by their respective interquartile ranges (IQRs). Differences in median intakes of food and nutrients and HEI-2015 scores were evaluated by Mann–Whitney test or Kruskal–Wallis test. Adjusted medians scores, as well as IQRs, were obtained from quantile regression. Quantile regression is used to adjust estimates of central tendency for distribution of highly skewed data^37^. Adjustment was made for socioeconomic factors including age, sex, educational status, and perceived financial status. All statistical analyses were performed with IBM SPSS Statistics for Windows, version 26.0 (IBM Corp., Armonk, NY, USA). This paper has reported results in accordance with STROBE-nut (Strengthening the Reporting of Observational studies in Epidemiology-Nutritional Epidemiology)^38^.

### Ethics approval and consent to participate

Approval for the research protocols and methodology was provided by the Ethical Committee of the Hungarian Scientific Council on Health (61327–2017/EKU). Participants gave their written informed consent in each study population in accordance with the Declaration of Helsinki and the Science Ethics Code of The Hungarian Academy of Sciences.

## Results

### Characteristics of the study participants

Table 1 summarizes the sociodemographic characteristics and nutritional status of the study participants. The study sample of the present analysis included 393 subjects from the HR settlements of Northeast Hungary and 415 subjects from HG population living in the same geographical area. The average age of participants was not statistically different between study groups (43.8 years and 43.0 years, respectively), but representation of females was higher among participants from HR settlements compared to HG group (73.3% vs. 55.7%). Educational level was significantly lower among HR population, with 85.7% of them having only 8 years of elementary school or less. More than half of the participants in both groups perceived their financial situation as fair, but there was a larger proportion of HR participants experiencing poor financial situation compared to HG participants (29% vs. 12%), while 31.8% of HG perceived it as good compared to 15.6% of HR. The nutritional status in both study groups was not significantly different as measured by average BMI values, but the distribution of nutritional status groups revealed a larger proportion of underweight and obese participants among HR.

**Table 1:** Characteristics of the study participants.

Variable ^a^	Hungarian Roma	Hungarian General	P*
Sex (females)	288 (73.3%)	231 (55.7%)	** < 0.001**
Age (years, x̄ (SD)	43.0 (13.0)	43.8 (12.6)	0.344
*Age group*			
18–34	114 (29.0%)	106 (25.5%)	0.643
35–44	88 (22.4%)	102 (24.6%)	
45–54	104 (26,5%)	107 (25.8%)	
55–70	87 (22.1%)	100 (24.1%)	
*Educational level*			
Elementary	336 (85.7%)	89 (21.5%)	** < 0.001**
Secondary	17 (4.3%)	90 (21.8%)	
Vocational training	38 (9.7%)	168 (40.7%)	
University degree	1 (0.3%)	66 (16.0%)	
Missing	1	2	
*Perceived financial status*			
Good	61 (15.6%)	129 (31.8%)	** < 0.001**
Fair	216 (55.1%)	229 (56.4%)	
Poor	115 (29.3%)	48 (11.8%)	
Missing	1	9	
*Nutritional status*			
BMI	27.7 (6.81)	27.2 (5.44)	0.308
Underweight	28 (7.1%)	11 (2.6%)	
Normal	124 (31.6%)	141 (34.0%)	
Overweight	97 (24.7%)	147 (35.4%)	** < 0.001**
Obese	144 (36.6%)	116 (28.0%)	

Data are presented as means (standard deviations) or numbers (percentages).

*Differences in means or percentage distributions were tested by t-test or chi-square test.

Educational level category had 3 missing values, perceived financial status had 10.

Significant values are in [bold].

### Food and nutrient intake used for HEI-2015 scores

Representative food and nutrient intake of HEI-2015 score components showed substantial differences between study groups, most of them statistically different (Table 2). Intakes of fruits, greens and beans, whole grains, seafood, and plant proteins (beans and nuts) were particularly low in both groups and statistically lower among HR participants. Median consumption of whole fruits and greens and beans was zero among HR and was significantly less than HG participants. The consumption of whole grains and seafood and plant proteins was generally very low, with zero median values in both groups and significantly lower values among HR. Intake of refined grains, SFAs, and sodium were high among all participants, but not statistically different between groups. Average SFAs intake was slightly above the recommended value of 10% of total energy intake. Daily sodium intakes were more than double of the recommendation. HR’s diet contained significantly more added sugar than HG’s while exceeding the recommended 10% of total energy intake.

**Table 2:** Food and nutrient intake used for Healthy Eating Index-2015 component scores.

Variable ^1^	Hungarian Roma	Hungarian general	P*
*Fruits*			
Total fruits^2^	2.58 (0.00–54.5)	37.72 (0.00–91. 35)	** < 0.001**
Whole fruit intake^3^	0.00 (0.00–45.05)	31.13 (0.00–81.21)	**0.000**
*Vegetables*			
Total vegetables^4^ Greens and beans^5^	127.266 (77.3–183.67) 0.00 (0.00–12.10)	135.99 (48.26–136.94) 4.83 (0.00–17.93)	0.160 ** < 0.001**
*Grains*			
Whole grains	0.00 (0.00–0.00)	0.00 (0.00–20.51)	** < 0.001**
Refined grains	84.00 (57.83–108.48)	81.74 (58.57–105.77)	0.546
Dairy^6^	38.71 (14.18–89.69)	63.46 (28.47–117.97)	** < 0.001**
*Protein Foods*			
Total protein foods^7^	98.32 (71.77–131.61)	108.18 (80.32–143.06)	**0.003**
Seafood and plant proteins^8^	0.00 (0.00–10.24)	3.60 (0.00–16.72)	** < 0.001**
*Fats*			
Fatty acids^9^	1.90 (1.61–2.22)	1.91 (1.67–2.28)	0.382
Saturated fats (% of energy)	10.47 (7.97–13.24)	10.58 (8.31–12.64)	0.678
Added sugars (% of energy)	11.56 (5.53–18.84)	6.69 (3.20–11.47)	** < 0.001**
Sodium	2.36 (1.99–2.86)	2.45 (2.01–2.89)	0.245

^1^Data represent average daily intake amounts in grams per 1000 kcal energy intake except in the cases indicated.

Values are medians (25th–75th percentiles).

^2^Includes 100% fruit juice.

^3^all forms except juice.

^4^Includes legumes (beans and peas).

^5^Includes legumes and dark-green leafy vegetables.

^6^Includes all milk products, such as fluid milk, yogurt, cheese, and fortified soy beverages.

^7^Includes meat, poultry, seafood, beans and peas, eggs, and nuts and seeds.

^8^Includes seafood, nuts, seeds, soy products (other than beverages), and legumes (beans and peas).

^9^Ratio of poly-and mono-unsaturated fatty acids (PUFAs and MUFAs) to saturated fatty acids (SFAs).

*Differences in the distribution of intakes were evaluated by Independent-Samples Mann–Whitney U test. Bold values represent significant differences.

### Healthy Eating Index-2015 scores of the study population

**Figure 1:**
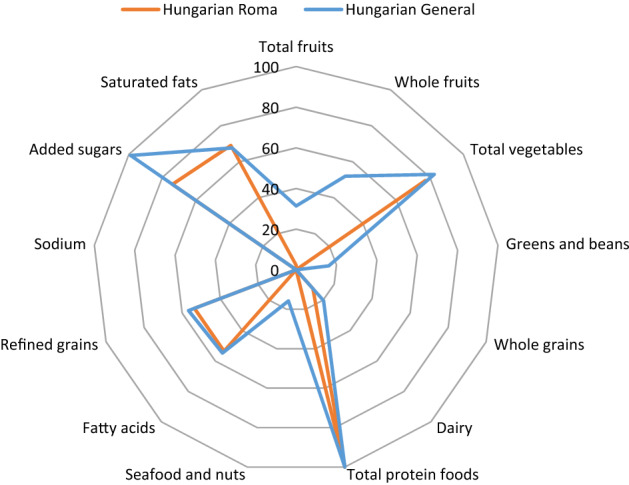
Radar plot of average Healthy Eating Index-2015 component scores for Hungarian Roma and general samples^*^. ^*^A radar plot displays information about each component score simultaneously. The outer edge of the “wheel” represents a score that is 100% of the maximum score for that component, while the centre of the circle represents a score of 0% of the score for any component.

Average HEI-2015 scores for HR and HG populations are presented in Table 3. Higher average scores represent overall better diet quality, and higher scores for adequacy components and for moderation components indicate better alignment to the current dietary recommendation. Figure 1 shows a radar plot of average Healthy Eating Index-2015 component scores for Hungarian Roma and general samples visualizing the multidimensional quality of the score. The median values of total HEI-2015 were below 50% of maximum points (100) and approximately 7 points less among HR, compared to HG participants (41.18 vs. 48.05). Adequacy of total fruits, whole fruits, greens and beans, whole grains and seafood, and plant proteins were particularly unfavorable among both groups, with significantly lower median scores among HR. Dairy scores were poor for both groups and significantly lower among HR. Total protein foods score was the only adequacy component which met the maximum score of 5 for most participants in both groups. Moderation components scores for sodium were generally very poor, with zero values for 75% of all participants and not different between HR and HG. Added sugar scored generally better in both groups but was less among HR. Scores for fatty acids and total SFAs were average and not different between groups.

**Table 3:** Average Healthy Eating Index-2015^1^ scores for Hungarian Roma and general samples.

Component ^a^	Maximum points	Hungarian Roma	Hungarian General	P*
Total HEI-2015 score	100	41.18 (33.23–48.35)	48.05 (39.13–56.41)	** < 0.001**
*Adequacy*				
Total fruits	5	0.11(0.00–2.27)	1.57 (0.00–3.81)	** < 0.001**
Whole fruits	5	0.00 (0.00–3.75)	2.60 (0.00–5.00)	** < 0.001 **
Total vegetables	5	3.86 (2.34–5.00)	4.13 (2.58–5.00)	0.129
Greens and beans	5	0.00 (0.00–2.02)	0.81 (0.00–2.99)	** < 0.001**
Whole grains	10	0.00 (0.00–0.00)	0.00 (0.00–4.82)	** < 0.001 **
Dairy	10	1.24 (0.46–2.88)	2.03(0.91–3.78)	** < 0.001**
Total protein foods	5	5.00 (5.00–5.00)	5.00 (5.00–5.00)	0.056
Seafood and plant proteins	5	0.00 (0.00–2.26)	0.79 (0.00–3.69)	** < 0.001**
Fatty acids	10	5.35 (3.19–7.82)	5.47 (3.62–8.34)	0.384
*Moderation*				
Refined grains	10	5.35 (3.19–7.83)	5.66 (2.28–8.94)	0.513
Sodium	10	0.00 (0.00–0.15)	0.00 (0.00–0.00)	0.889
Added sugars	10	7.40 (3.67–10.0)	9.90 (7.45–10.0)	** < 0.001**
Saturated fats	10	6.91 (3.44–10.0)	6.77 (4.20–9.62)	0.848

^1^The HEI-2015 is a measure of diet quality and includes 13 components that capture the balance among food groups including those to encourage (adequacy components) and those for which there are limits (moderation components). Higher scores of adequacy components reflect higher intakes that meet the standards. Higher scores of moderation components indicate lower intakes that are more desirable. Intakes between the minimum and maximum standards are scored proportionately.

^a^ Values are medians (25th-75th percentiles). Median values of components may not add up to median total HEI scores.

*Differences in median scores were evaluated by Independent-Samples Mann–Whitney U test. Bold values represent significant differences.

### Stratified analyses of HEI-2015 scores

We performed a stratified analysis of total HEI-2015 scores to explore differences in HEI-2015 scores while accounting for sociodemographic characteristics (Table 4). The stratified analysis revealed no differences between sexes. Total-HEI-2015 was gradually better scored among older age groups but did not significantly differ among HR. Individuals with higher educational level had better total HEI-2015 scores among the HG population, but not in case of HR. Self-perceived financial status was inconsistently associated with total HEI-2015 scores and showed higher scores among those HR who perceived their financial status as worse. Individuals belonging to underweight and normal nutritional status had lower total HEI-2015 scores than those belonging to overweight or obesity categories among the HR, but not among HG.

**Table 4:** Average Total Healthy Eating Index 2015 scores by sex, age, educational status, perceived financial situation, and BMI categories.

Component *	All participants ^a^	Hungarian Roma ^b^	Hungarian general ^c^	P**
Total HEI-2015 score	43.84 (36.78–52.81)	41.18 (33.23–48.35)	48.05 (39.13–56.41)	** < 0.001**
*Sex*				
Male	43.01 (35.50–51.85)	39.96 (31.84–45.86)	46.55 (38.20–54.29)	0.072 ^a^
Female	44.37 (37.26–53.33)	41.72 (33.90–49.08)	49.52 (39.58–54.29)	**0.021 **^**b**^
				**0.006 **^**c**^
*Age*				
18–34	42.78 (34.75–53.27)	39.50 (32.62–47.00)	49.28 (37.28–56.90)	**0.025 **^**a**^
35–44	42.95 (37.08–49.94)	41.36 (33.66–47.20	44.07(38.62–51.30)	0.107 ^b^
45–54	44.04 (37.33–53.32)	40.58 (31.39–47.76)	48.33 (39.96–56.97)	**0.026 **^**c**^
55–70	46.65 (38.53–54.17)	43.20 (35.45–49.71)	50.34 (40.16–58.67)	
*Educational levels*				
Elementary	41.69 (33.99–49.14)	41.45 (33.17–48.61)	43.66 (37.20–51.58)	** < 0.001**^a^
Secondary	46.56 (39.03–53.94)	38.97 (32.62–42.07)	48.84 (41.84–57.42)	0.214 ^b^
Vocational training	44.37 (37.50–54.34)	41.86 (33.35–49.36)	46.18.(37.84–55.95)	** < 0.001**^**c**^
University degree	56.14 (49.69–59.77)	–	55.90 (49.60–60.04)	
*Perceived financial status*				
Poor	43.39 (37.18–52.46)	43.38 (36.39–51.33)	42.07 (37.21–54.45)	0.701 ^a^
Fair	44.07 (37.17–52.42)	40.66 (33.10–47.16)	48.84 (40.29–56.48)	**0.008 **^**b**^
Good	44.02 (36.20–54.40)	36.43 (31.74–46.33)	48.39 (38.52–57.14)	0.240 ^c^
*Nutritional status*				
Underweight	39.31 (29.76–45.74)	35.49 (27.19–43.14)	48.39 (42.39–55.92)	**0.002 **^**a**^
Normal	42.95 (35.27–50.99)	39.96 (31.88–46.16)	46.35 (37.72–56.42)	**0.004 **^**b**^
Overweight	45.98 (37.68–54.36)	42.10 (33.93–50.32)	48.33 (38.71–57.60)	0.573 ^c^
Obese	44.03(37.79–52.46)	42.25 (34.33–49.52)	49.49 (40.97–55.44)	

*Values are medians (25–75th percentiles).

**Differences in distribution of scores were evaluated by Mann–Whitney or Kruskal–Wallis test. Bold values represent significant differences between socioeconomic variables within groups of all participants^a^, Hungarian Roma^b^, and Hungarian General^c^.

### Adjusted median HEI-2015 scores

Results of quantile regression for total median HEI-2015 and its components scores, adjusted for age, sex, educational status, and perceived financial status for both HR and HG populations are presented in Table 5. Overall, there were little changes observed in adjusted median scores compared to the unadjusted ones. Differences between HR and HG remained similar and maintained the same direction. Adjusted median scores for whole grains, total protein foods, and sodium were not significantly different. All other adjusted median scores showed significantly lower values for HR than HG.

**Table 5:** Estimated median Healthy Eating Index-2015 scores adjusted for age, sex, educational status, and perceived financial status by quantile regression.

Component^a^	Maximum points	Hungarian Roma	Hungarian general	*P*^b^
Total HEI-2015 score	100	41.64 (39.46–42.80)	47.20 (45.69–51.09)	** < 0.001**
*Adequacy*				
Total fruits	5	0.10 (0.05–0.46)	1.71 (1.02–2.33)	** < 0.001**
Whole fruits	5	0.00 (0.00–0.00)	2.83 (1.63–3.86)	** < 0.001**
Total vegetables	5	4.14 (3.41–4.36)	4.18 (3.81–4.57)	** < 0.001**
Greens and beans	5	0.00 (0.00–0.00)	0.95 (0.36–1.17)	** < 0.001**
Whole grains	10	0.00 (0.00–0.00)	0.00 (0.00–0.00)	1.000
Dairy	10	1.41 (0.99–1.62)	1.86 (1.48–2.43)	** < 0.001**
Total protein foods	5	5.00 (5.00–5.00)	5.00 (5.00–5.00)	1.000
Seafood and plant proteins	5	0.00 (0.00–0.00)	0.58 (0.42–1.07)	** < 0.001**
Fatty acids	10	5.34 (5.07–5.71)	5.43 (5.06–5.78)	**0.004**
*Moderation*				
Refined grains	10	5.56 (4.80–5.87)	5.97 (5.31–6.62)	** < 0.001**
Sodium	10	0.00 (0.00–0.00)	0.00 (0.00–0.00)	1.000
Added sugars	10	7.67 (6.39–8.06)	9.80 (8.57–10.0)	** < 0.001**
Saturated fats	10	6.88 (6.17–7.28)	6.94 (6.63–7.36)	**0.018**

^a^Values are medians (25th–75th percentiles). Median values of components may not add up to median total HEI scores.

^b^Differences in median scores were evaluated by Independent-Samples Mann–Whitney U test. Bold values represent significant differences.

## Discussion

In this report, we evaluated diet quality for a representative sample of Roma minority living in segregated colonies in Northeast Hungary, while comparing that of a HG sample. Overall, our evaluation indicated poor alignment to current dietary guidelines based on HEI-2015 total and component scores among both study groups (i.e., HR and HG), with HR often having worse scores.

Significant differences were found between HR and HG with regards to HEI-2015 scores in the adequacy of fruits, greens and beans, whole grains, seafood, and plant proteins and their intake was substandard among both groups, with HR having significantly lower scores. Lower overall socioeconomic status was seen among Roma minority sample compared to the HG group, but these discrepancies did not display any consistent association with dietary quality. The presence of educational and financial differences may suggest that drivers of poor diet quality can be attributed to the food environment rather than to the socioeconomic characteristics.

Diet is one of the main contributors to disparities in many diet-related NCDs among different populations, including ethnic groups. Several epidemiological studies have confirmed that diet quality follows a socioeconomic gradient, with the more disadvantaged groups having lower consumption of health-promoting food groups such as whole grains, lean meats, fish, low-fat dairy products, fresh vegetables and fruits, and higher consumption of refined grains and animal fats^39–41^. Overall, there is a lack of health research among disadvantaged populations and very few studies have been conducted for the Roma^5^.

The quality of diet and adherence to healthy dietary patterns was low among both groups and frequently worse among the HR. Adequacy component scores for fruits, dark green vegetables and beans, whole grains, seafood, and plant proteins reflected minimal consumption of those food groups in almost all participants. These scores were all significantly lower among the HR population compared to HG population. The median total and whole fruits scores were zero among the HR population, considering that 50% did not have a daily regular fruit intake, compared to 30% of the HG sample. Whole grains scores were one of the most unfavorable since 80% of the HR population and 50% of the HG group did not have any recorded intake of whole grain products. Similar low scores reflected minimal or no consumption of beans, dark green vegetables, nuts, and fish. Fatty acids scores (the ratio of poly- and mono-unsaturated fatty acids to SFAs) were approximately 5 to 10 in both groups, clearly indicating a higher share of SFAs. The only adequacy component that met the maximum value in more than 75% of all participants was the total ‘protein foods’ score, although this is partly originated from the intake of red meat and processed meat. Such food intake has been convincingly associated with increased risk of colorectal cancer (CRC)^42^. CRC is a major health problem in Hungary, which has been reported to be the country with the highest age-standardized death rate per 100,000 population from CRC in the world^43^.

High intakes of refined grains and salt were mirrored in the moderation component scores. Added sugar scores looked better, but this might be due to general underreporting of sugar-sweetened beverages and confectionery. Average SFAs scores indicated that half of the study participants had more than the recommended 10% of total energy derived from saturated fatty acid intake, and they did not differ between HR and HG. The analysis of sociodemographic factors revealed that lowest educational level and poor perceived financial situation were associated with lowest total HEI-2015 scores in the total sample and HG but not in the case of HR. We observed similar differences in diet quality between HR and HG after adjustment of HEI-2015 scores to age, sex, educational level, and perceived financial status. This may suggest an independent effect of ethnicity on diet quality, which can arise from stronger attachment to traditional Eastern-European diet, characterized by high amounts of white bread and refined grain products, preserved vegetables, processed meat, and lard^22^.

Dietary patterns encompass food and nutrient consumption and have a better predictive value for diet-related NCDs risk, compared to individual foods or nutrients. Dietary guidelines highlight the importance of overall dietary patterns in the prevention of chronic diseases. Studying dietary patterns in relation to morbidity and mortality from chronic diseases is a competent and efficient method to evaluate the health effects of adherence to dietary guidelines^44–46^. Several countries have developed food based dietary guidelines (FBDGs) to promote health and prevent chronic diseases in their population. The dietary guidelines in Hungary followed the international food-based recommendations, the latest version was developed by the Hungarian Dietetic Association after the introduction of the American Dietary Guidelines in 2015, using an icon similar to MyPlate^47^. Results of a recent systematic review on the adherence to FBDGs across countries found that almost 40% of populations in both high-income and low-income countries did not adhere to their national FBDGs^48,49^.

The association of diet quality assessed by HEI-2015 and similar scores, with the risk of NCDs has been evaluated by several follow-up cohort studies. An updated systematic review and meta-analysis of cohort studies found that high-quality diets were associated with a significant reduction in the risk of all-cause mortality, CVDs, cancer, T2DM, and neurodegenerative disease by 22%, 22%, 16%, 18%, and 15%, respectively^50^.

The socioeconomic status, ethnicity, as well as perceived economic barriers, and perceived nutritional benefits all affect the dietary intake of individuals. Better scores for healthy eating have been consistently observed in higher socioeconomic populations, while scores for less healthy diet in lower socioeconomic populations across different countries^41,51–55^.

Such findings and our results are in line with our previous dietary analyses^24,25^, which evaluated the macro-and micro-nutrients intake and their adequacy in relation to international recommended intake values for both HR and HG. In one of our previous studies, results indicated overall poor nutrient patterns for both groups, and nutrient profile of participants from Roma settlements had lower odds for achieving population nutrient intake goals^24^. In another work, we used nutrient-based scoring and adjusted regression models but observed no notable effect of Roma ethnicity on nutrient-based dietary patterns, except for the Dietary Inflammatory Index^25^.

In the present analysis diet quality and adherence to dietary guidelines, as assessed by HEI-2015 scores, was alarmingly poor among both HR and HG samples, but considerably worse among HR. The values of individual component scores for fruits, greens and beans, whole grains, seafood, and plant proteins were extremely poor among participants and significantly lower among the HR population. Such observed poor diet quality has the potential to contribute to an increased risk of CVDs, which has been described previously in the same population^13^.

Previous results of comparative health surveys among inhabitants of Hungarian Roma settlements found that their health was poor and comparable to the health status of people in the lowest socioeconomic quartile of the general population^7^. The socioeconomic conditions of the Roma may explain their worse health status to a certain extent, but only partially determined their less healthy behaviors, indicating the effect of cultural differences^56^. A critical evaluation of health policies to integrate Roma communities into the European region has shown that there are many obstacles and that a comprehensive policy framework needs to be developed, including education, economic, labor market, housing, and environmental and territorial development policies^57^.

An investigation on the effects of the health policies implemented in the framework of the Decade of Roma Inclusion program showed that Roma remained severely disadvantaged as compared to the general population^58^. Compared to the general Hungarian population, the gap had narrowed with regards to employment, perceived financial status, utilization of primary health care, smoking, and daily consumption of fruits and vegetables, but widened with regards to education, self-reported health status, the use of secondary health care services and dental care, overweight and alcohol consumption. The association between deteriorated diet quality among HR and increased social disadvantage highlights the need for specifically targeted interventions among HR. The current Hungarian national strategy for the Roma does not specifically target the Roma ethnic minority, but all vulnerable groups, but health and nutrition policy is missing from these documents^59^.

Although our study provides a comprehensive dietary pattern analysis of the Roma populations living in segregated colonies there are some limitations that need to be considered. Dietary measurement was based only on two days that may not capture the seasonal and long-term variations of the usual diet. There is a possibility that self-reported dietary intake provides biased results. Participants can forget to report some consumed foods (recall bias) or underreport their intake of well-known unhealthier foods and over-report their intakes of healthier foods (social desirability bias). Like any measure of a construct such as diet quality, the HEI-2015 has limitations. In applications of self-report dietary intake data, such as our 24-h recall, it is important to consider the influence of measurement error in the interpretation of HEI-2015 scores. Different dietary components appear to be misreported to different degrees^60,61^. To the extent that less healthy foods may be underreported, and more healthy foods may be over-reported, HEI scores based on self-report intake data may be overly favorable. The latter may mean that the current dietary situation maybe even worse than described. Another consideration, when using the HEI-2015 is that there can be multiple ways to arrive at the same total score (a score can be attained through very different profiles of component scores). To address this, we examined component scores, as well as total scores. In general, there is greater confidence regarding total scores at the higher and lower ends of the range of scores because they represent more homogenous diets across individuals. In addition, unlike the range of intakes for nutrients or food groups, HEI-2015 component, and total scores are truncated, and so may not capture some important information. For example, a high score for ‘total protein foods’ does not capture potentially excessive intakes, which could be further explored. Another important limitation is that the HEI-2015 has not yet been adapted and validated for the Hungarian population. HEI-2015 is based on the 2015–2020 Dietary Guidelines for Americans and not be entirely applicable to non-American populations due to existing differences between populations or cultures that might have significant variations in common foods consumed^33,62–64^.

Our sample is only representative for Roma populations living in segregated colonies in Northeast Hungary, but not representative for the whole Roma population in Hungary. The Roma ethnicity status was also self-reported, but the identification was promoted by participation of Roma university students during the data collection process. The HG sample may include individuals of Roma ethnicity that can contribute to the underestimation of differences between the study groups.

## Conclusions

In summary, we report differences in diet quality, operationalized as the HEI-2015, with the Roma ethnic minority having extremely poor and frequently worse scores compared to the HG population living in the same geographical area. To date, this is the first study in Hungary and elsewhere that uses food-level intake data to assess dietary quality, as well as adherence to food-based dietary guidelines among Roma minority. Our findings highlight a potentially increased risk for diet-related NCDs, that may particularly arise from inadequate intakes of important food groups. Future health intervention programs should focus on addressing diet disparities of ethnic groups and choose appropriate strategies that accommodate the ethnic and cultural differences of the Roma and consider their socio-economic barriers.

## Data Availability

The datasets used and/or analyzed during the current study are available from the corresponding author on reasonable request.
